# The Preoperative Diagnostic Value of MRI and Otoneural Tests in Acoustic Neuroma

**DOI:** 10.3389/fonc.2021.626485

**Published:** 2021-06-29

**Authors:** Qingqing Dai, Meijun Zheng, Qiurong Chen, Hong Zheng, Bilan Li

**Affiliations:** ^1^ Department of Otolaryngology, West China Hospital of Sichuan University, Chengdu, China; ^2^ Department of Otolaryngology-Head and Neck Surgery, Sichuan Provincial People’s Hospital, University of Electronic Science and Technology of China, Chengdu, China

**Keywords:** acoustic neuroma, magnetic resonance imaging, audiometry, vestibular, examination, ONT

## Abstract

**Objectives:**

To determine the preoperative diagnostic accuracy of MRI and otoneural tests (ONT) for acoustic neuroma (AN) in a cohort of unselected patients with pontocerebellar angle tumors. To find a convenient way to screening out the potential asymptomatic AN patient earlier.

**Design:**

This diagnostic accuracy study was performed in a central hospital and included a consecutive sample of unilateral incipient pontocerebellar angle tumor patients referred for MRI and ONT before surgery. Different AN features of MRI and ONT were collected and concluded into preoperative diagnostic variables or variable combinations. Those of MRI and ONT are analyzed and compared with biopsy results by multivariable receiver operating characteristic (ROC) analysis. The early-stage group, the course of which is 1 year or less, was separately computed and compared.

**Results:**

Eighty-three subjects were collected from June 2013 to June 2019; 62 were confirmed AN postoperatively by biopsy, whereas others are not AN. The area under the curve (AUC) of MRI was 0.611, whereas the AUC of ONT was 0.708. In the early-stage group, the AUC of MRI was 0.539, and the AUC of ONT was 0.744.

**Conclusions:**

ONT was able to identify more subjects affected by unilateral incipient AN than MRI preoperatively. Given that ONT is a functional test for internal auditory canal nerves, it is an optimal screening test for AN patients because it provides more information than MRI for the further clinical plan. It is particularly noteworthy for identifying asymptomatic AN patients and for early stage. Therefore, it may help more patients from unnessesary surgery. Furthermore, an MRI follow-up is suggested if the patient was found alert in ONT.

## Introduction

Acoustic neuroma (AN), also termed vestibulocochlear schwannoma, from the Schwann cells of the vestibular division or the cochlear division, is the most common tumor in pontocerebellar angle. AN is shown to represent 6% to 8% of all intracranial tumors (1). The typically presenting complaints in AN patients are unilateral hearing loss, tinnitus, and disequilibrium. These disorders do not necessarily correlate close with tumor size or shape (2). As the natural history of the AN is variable (3), only less than 1% of AN exhibit sufficient growth to become clinically active. The patients with small tumors that do not compress the nerves and lead dysfunction may be asymptomatic. Thus, AN patients’ managing options vary from observation, microsurgery, stereotactic radiosurgery to surgical debulking (4).

Nevertheless, it is an arduous task to differentiate AN from other tumors in the pontocerebellar angle before surgery (5). So the vital part for optimizing management, early diagnosis, is still under improvement. According to the guidelines of AN diagnosis, magnetic resonance imaging (MRI) will be performed to confirm the diagnosis after otoneural tests (ONT) (6, 7). Thus, ONT and MRI have assumed a significant role in the diagnosis of AN.

Otoneural tests (ONT) usually include audiological and vestibular examinations. Audiometry of AN is often characterized by unilateral or asymmetric sensorineural hearing loss and poor speech recognition (8), whereas vestibular examinations often found abnormal vestibular-ocular reflex (VOR), or asymmetric caloric test, or spontaneous nystagmus (9).

MRI is currently the standard diagnostic test for AN besides pathology (10). With the extensive application of MRI, the early diagnosis rate of AN has been improved gradually. However, not all patients show the typical clinical traits of MRI in AN. Furthermore, as tumor size reduces, the sensitivity and specificity of this golden standard before surgery declines (11).

Because MRI is not sufficient enough, can we dig out more information from routine ONT tests? Besides the function of screening, are hearing and vestibular examinations helpful for the qualitative diagnosis of AN? This study aimed to assess the diagnostic accuracy of ONT for AN in a cohort of unselected pontocerebellar angle tumor patients. We studied the MRI and ONT tests that are commonly used in daily clinical practice, which are as follows: pure-tone test, speech reception threshold (SRT), speech discrimination score (SDS), videonystagmography (VNG), and caloric test. Moreover, we creatively comprehended the ONT tests to make out the score easily to diagnose and determine the preoperative diagnostic accuracy of MRI and ONT for AN.

## Materials and Methods

This work was designed as a preoperative diagnostic accuracy study using otoneural tests in a cohort of unselected patients with pontocerebellar angle tumors. The samples were collected prospectively among patients referred for vestibular testing at the balance laboratory of our department. We examined the medical records of all patients diagnosed with unilateral incipient pontocerebellar angle tumors based on their MRI imaging between June 2013 and June 2019 in the neurosurgery department at West China Hospital.

All the included patients were required to have otoneural tests and MRI examinations 1 week before surgery. The machine used in this study for ONT is the Ulmer VNG infrared nystagmus view system (Synapsys, France). Every patient has also undergone an enhanced MRI scan of the head in West China Hospital.

Complete otoneural tests consist of the following hearing function tests: pure tone audiometry, acoustic-conductivity resistance test, speech reception threshold (SRT), speech discrimination score (SDS); and vestibular function tests under VNG: VOR tests (including saccade test, smooth pursuit test, optokinetic test, gaze test, and spontaneous nystagmus), and caloric test. Patients with multiple neurofibromatosis and recurrent cerebellopontine angle tumor; or patients with visual impairment, severe systemic diseases, otitis media, or limited ocular movement who failed to complete the test were excluded from this study. All candidates were given informed consent following all the guidelines for investigation with human subjects required by the ethics committee of our hospital. The patients who agreed to sign it were included in the study.

Ultimately, 83 patients with unilateral incipient pontocerebellar angle tumors were included. Different AN features of MRI and ONT were collected and concluded into preoperative diagnostic variables or variable combinations. Those of MRI and ONT are analyzed and compared with after-surgery pathological results by multivariable receiver operating characteristic (ROC) analysis.

Variables of interest were as follows:

1. The final pathological result after surgery (pathol. for short): defined as the actual fact. AN for positive (1), other tumors for negative (0).
2. MRI result before surgery (MRI for short): If there is enlargement/enhancement of the inner auditory canal defined by experienced radiologists as positive (1), otherwise for negative (0).
3. Audiometry before surgery (speech. for short): acoustic-conductivity resistance test must be normal. So this variable includes pure-tone test, speech reception threshold (SRT), and speech discrimination score (SDS). 10 dBHL or more neural hearing impairment or speech discrimination impairment for positive (1). All tests are normal for negative (0).
4. VOR tests before surgery (oculom. for short): including saccade test, smooth pursuit test, optokinetic test, gaze test, and spontaneous nystagmus. If the VOR is impaired, or spontaneous nystagmus, or central positional nystagmus are defined as positive (1), all normal for negative (0).
5. Caloric test before surgery (Calori. for short): affected side impaired (UW>15%) for positive (1), normal for negative (0). Our vestibular laboratory has set our own normal standard by recruiting normal local healthy control, and then set the UW >15% as abnormal.
6. Vestibular function impairment before surgery (vestib. for short): If there are two positives in VOR, nystagmus, or caloric test is defined as positive (1), all normal or only one positive for negative (0).

In this way, we transferred all results into binary variables.

We divided the patients into early-stage and late-stage groups by the course time of 1 year to mining more information about the early diagnosis of AN.

Statistical analysis was carried out with software MedCalc, version 13.0. General data are described by constituent ratios. Multiparametric receiver operating curve (ROC) analysis was adopted to achieve diagnostic accuracy. The methodology is by DeLong et al. (1988) and Binomial exact Confidence Interval for the AUC.

## Results

### Patient Characteristics

Finally, 25 males and 58 females are recruited, sex ratio is 1:2.32. The average age is 48 years (17–75 years), and the average course is 3.1 years (1 month to 20 years) (**Table 1**). Forty-five cases are left side affected, side ratio is 1.18: 1, left-sided are slightly more than the right-sided. AN was found in 60 cases (72.29%). Other tumors include 14 meningiomas (16.87%) and 9 other types (10.84%) of pontocerebellar angle tumor (two cases of arachnoid cyst, two cases of epidermoid cyst, one case of ependymoma, one case of hemangioma, one case of hemangioblastoma, one case of trigeminal neuroma, and one case of poorly differentiated carcinoma).

**Table 1:** General data of age and course of all patients.

	Age (year)	Course (year)
Lowest value	17	0.1
Highest value	74	20
Arithmetic mean	47.95	3.08
95% CI for the mean	44.9774 to 50.9304	2.1488 to 4.0078
Median	48.56	1
Shapiro-Wilk test for normal distribution	W=0.98	W=0.67
	Accept normality (P=0.3007)	Reject normality (P<0.0001)

### Diagnostic Results of the Variables

**Table 2** shows the diagnostic results of different tests for AN/non-AN groups. Audiometry seems to have the highest sensitivity for AN.

**Table 2:** Positive results of different tests in AN/non-AN groups.

Group	pathol	MRI	speech	oculom	calori	vestib
AN	60	42	56	33	47	51
non-AN	23	11	10	4	12	10

pathol, the final pathological result after surgery; MRI, MRI result before surgery; speech, audiometry before surgery; oculom, including saccade test, smooth pursuit test, optokinetic test, gaze test, and spontaneous nystagmus; calori, caloric test before surgery; vestib, vestibular function impairment before surgery.

### Diagnostic Accuracy

#### ROC for Variables’ Comparison

**Figure 1:**
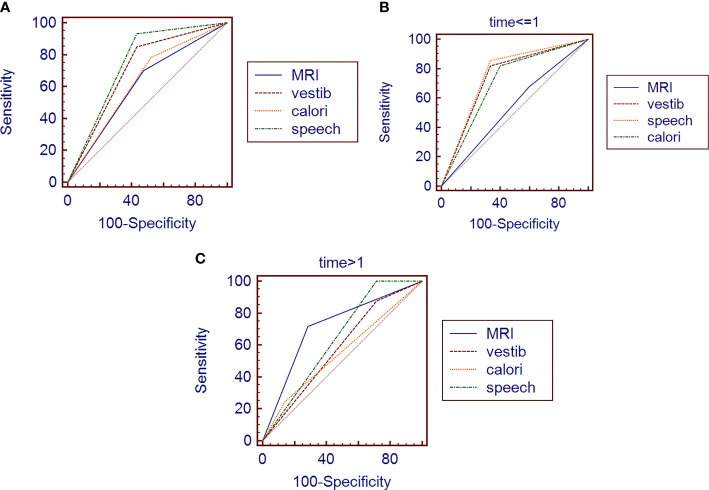
ROC of different variables in general **(A)** /in early-stage group **(B)** /in late-stage group **(C)**. MRI, MRI result before surgery; vestib, vestibular function impairment before surgery; calori, caloric test before surgery; speech, audiometry before surgery time, the course of disease (we divided the patients into early-stage and late-stage groups by the course time of one year).

Assuming an AN diagnosis based on the abovementioned single tests, ROC analysis revealed that no test achieved an accuracy (AUC ≥ 0.75) suitable for clinical use (**Table 3**), even though vestib. is a combination of oculom. and calori. tests. The highest diagnostic accuracy was obtained from audiometry (**Figure 1A**).

**Table 3:** AUC of different variables in general/in early-stage group/in late-stage group.

		AUC	Standard Error ^a^	95% CI ^b^
In general	MRI	0.611	0.0610	0.498 to 0.716
	vestib	0.708	0.0577	0.598 to 0.802
	calori	0.631	0.0596	0.518 to 0.734
	speech	0.749	0.0553	0.642 to 0.838
In early-stage group	MRI	0.539	0.0794	0.381 to 0.692
	vestib	0.744	0.0730	0.588 to 0.865
	speech	0.762	0.0714	0.608 to 0.878
	calori	0.711	0.0751	0.552 to 0.839
In late-stage group	MRI	0.717	0.101	0.550 to 0.849
	vestib	0.580	0.0969	0.412 to 0.736
	calori	0.554	0.0813	0.386 to 0.713
	speech	0.643	0.0922	0.474 to 0.789

a DeLong et al., 1988.

b Binomial exact.

MRI, MRI result before surgery; vestib, vestibular function impairment before surgery; calori, caloric test before surgery; speech, audiometry before surgery.

#### ROC for Early-Stage Group

In the group of patients whose course is less than 1 year, MRI (AUC = 0.539) is much less sensitive than the functional tests (**Table 3**). The greatest accuracy (AUC = 0.762) for early-stage AN is still based on audiometry (**Figure 1B**), but the combination of vestibular function tests are all good (AUC > 0.7).

#### ROC for Late-Stage Group

In the group of patients whose course is more than 1 year, MRI (AUC = 0.717) is much better than the functional tests (**Table 3**). The sensitivity of MRI for late-stage AN is still not satisfactory, but its specificity is much better than the functional tests this time (**Figure 1C**).

## Discussion

As the retro-cochlear tumors in internal acoustic meatus, such as AN, can compress the vestibular nerve, there are further deterioration in the functions of nerves and diminishing of the blood supply of the inner ear (12). Therefore, AN should be detectable with the audial and vestibular tests and may be more sensitive (13). Both hearing and vestibular function tests are valuable for the diagnosis of AN (14). We usually call these tests altogether as otoneural tests.

In this study, we did not analyze the role of vestibular-evoked myogenic potentials (VEMP) in the diagnosis because VEMPs are not usual routine tests. Sometimes patients fail to produce VEMPs bilaterally, rendering these tests inconclusive, especially when testing oVEMP (15). Although increasing attention has been focused on the role of VEMP in the assessment of patients with AN, there is no consensus about the use of VEMPs in detecting AN (14), maybe because the positive rate of VEMP is highly related to the tumor size (12). Moreover, researchers found that VEMP can be normal with tumors under 1.5 cm (16).

MRI is required in patients with positive ONT results. Of course, MRI is still advisable even if the otoneural tests are negative (6). Although the progress of MRI made it possible to diagnose smaller and asymptomatic tumors than were previously, after a pontocerebellar angle tumor is found from MRI, an optimal treatment plan will be produced. However, different kinds of pontocerebellar angle tumors require different management (4, 17), so the specificity of diagnosis is vital for AN management. Sometimes, it can be tough to differentiate AN from other symptoms because the symptoms vary (18). It is reported that AN’s symptoms may relate to the tumor characteristics (tumor size or distribution), patient age or gender, and also the medical level (2). Therefore, predicting the potential diagnoses, depending on the clinical features, is not relivable. Nevertheless, the signs of AN in MRI are unsatisfactory, especially in patients with similar clinical and radiological features (10). The qualitative diagnosis of AN from MRI is only approximately 70% (18), which is similar to our result (71.7% in the late group).

Furthermore, there are similar shortcomings of radiological examinations in AN diagnosis: the low specificity (**Figure 1A**), especially in the early stage (**Figure 1B**), because the smaller tumors are usually asymptomatic, making it complicated for early diagnosis. Some researchers already thought about this misdiagnosis and tried to use a hearing test to help the diagnostic value of MRI (19). However, adding a new test is a new burden for the economy and time-consuming.

Patients’ initial examinations are always functional signs using ONT tests before MRI (7), maybe because most of the AN patients visited the otolaryngology department because of hearing loss, tinnitus, speech resolution, and other symptoms (5). Therefore, we may use these data to help differentiate AN from other pontocerebellar angle tumors. However lots of papers found that the significance of a single test for diagnosis is limited. We creatively combined them as a parameter to help diagnose, and our study shows the high accuracy of ONT in detecting AN among patients with pontocerebellar angle tumors, especially for the early stage. This is of clinical relevance, considering that most AN are found from MRI as pontocerebellar angle tumors but were only diagnosed after surgery. However, as it is hard for us to remove the tumors without any complications, even for experienced surgeons, surgery is not always the best choice for AN (20). Hence, the importance of ONT accuracy for AN is further emphasized because it may help avoid misdiagnosis and unnecessary surgery or suboptimal treatment.

On the other hand, this result may suggest an MRI follow-up if the patient was found alert in ONT.

## Conclusions

Among the lesions found by MRI in the pontocerebellar angle, preoperative ONT was able to get further insight into AN’s features and identify more subjects affected by unilateral incipient AN than preoperative MRI. Given that ONT is a routine functional test for internal auditory canal lesions, it is an optimal screening test for AN patients and guides in the follow-up treatment. It is particularly noteworthy for identifying early-stage AN patients, given that it provides meaningful guidance for reducing operation rate, guiding radiotherapy, evaluating prognosis, and improving patients’ quality of life.

## Data Availability Statement

The raw data supporting the conclusions of this article will be made available by the authors, without undue reservation.

## Ethics Statement

The studies involving human participants were reviewed and approved by the Ethics Committee of West China Hospital. The patients/participants provided their written informed consent to participate in this study.

## Author Contributions

MZ, QD, BL, and HZ carried out the study. QD, MZ, and HZ conceived the study and participated in its design and coordination. QD, MZ, and BL drafted the manuscript. QC, QD, and MZ participated in patient collecting and data works. QC, QD, and BL performed the statistical analyses. All authors contributed to the article and approved the submitted version.

## Funding

This study was supported by the Chinese Twelfth Five-Year National Science and Technology Support Program project (project number: 2012BAI12B00).

## Conflict of Interest

The authors declare that the research was conducted in the absence of any commercial or financial relationships that could be construed as a potential conflict of interest.
